# Robson's Ten Group Classification System to Evaluate Cesarean Section Rates in Honduras: The Relevance of Labor Induction

**DOI:** 10.1055/s-0042-1753547

**Published:** 2022-10-10

**Authors:** Lester David Castro Paz, Rigoberto Castro Banegas, Adriana Gomes Luz, Maria Laura Costa

**Affiliations:** 1 Department of Obstetrics, University of Campinas, Campinas, SP, Brazil; 2 Department of Obstetrics and Gynecology, Hospital Dr. Roberto Suazo Cordova, La Paz, Honduras

**Keywords:** cesarean section, labor, induced, childbirth, cesárea, trabalho de parto, indução, parto

## Abstract

**Objective** To use the Robson Ten Group Classification (RTGC) to analyze cesarean section (CS) rates in a Honduran maternity hospital, with focus in groups that consider induction of labor.

**Methods** Cross-sectional study. Women admitted for childbirth (August 2017 to October 2018) were classified according to the RTGC. The CS rate for each group and the contribution to the overall CS rate was calculated, with further analyses of the induction of labor among term primiparous (group 2a), term multiparous (group 4a), and cases with one previous CS (group 5.1).

**Results** A total of 4,356 women were considered, with an overall CS rate of 26.1%. Group 3 was the largest group, with 38.6% (1,682/4,356) of the cases, followed by Group 1, with 30.8% (1,342/4,356), and Group 5, with 10.3% (450/4,356). Considering the contribution to overall CS rates per group, Group 5 contributed with 30.4% (345/1,136) of the CSs and within this group, 286/345 (82.9%) had 1 previous CS, with a CS rate > 70%. Groups 1 and 3, with 26.6% (291/1,136) and 13.5% (153/1,136), respectively, were the second and third larger contributors to the CS rate. Groups 2a and 4a had high induction success, with low CS rates (18.4 and 16.9%, respectively).

**Conclusion** The RTGC is a useful tool to assess CS rates in different healthcare facilities. Groups 5, 1, and 3 were the main contributors to the CS rate, and groups 2 and 4 showed the impact and importance of induction of labor. These findings may support future interventions to reduce unnecessary CS, especially among primiparous and in women with previous CS.

## Resumo

**Objetivo** Utilizar a Classificação de Dez Grupos de Robson (RTGC, na sigla em inglês) para analisar as taxas de cesárea (CS, na sigla em inglês) em uma maternidade hondurenha.

**Métodos** Estudo de corte transversal em uma maternidade em Honduras. As mulheres internadas para o parto (agosto de 2017 a outubro de 2018) foram classificadas segundo a RTGC. Calculou-se a taxa de CS para cada grupo e a contribuição para a taxa geral de CS, com análises adicionais da indução do trabalho de parto entre as primíparas a termo (grupo 2a), multíparas a termo (grupo 4a) e casos com uma CS anterior (grupo 5.1).

**Resultados** foram consideradas 4.356 mulheres, com uma taxa geral de CS de 26.1%. O Grupo 3 foi o maior grupo, com 38,.6% (1.682/4.356) dos casos; seguido pelo Grupo 1, com 30,8% (1.342/4.356), e pelo Grupo 5, com 10,3% (450/4,356). Considerando a contribuição para as taxas globais de CS por grupo, o Grupo 5 contribuiu com 30,4% (345/1,136) das CS, dos quais 286 (82.9%) tinha uma CS anterior, com um índice de CS > 70%. Os grupos 1 e 3, com 291/1.136 (26.6%) e 153/1.136 (13,5%), respectivamente, foram o segundo e terceiro maiores contribuintes para a taxa de CS. Os grupos 2a e 4a tiveram alto sucesso de indução, com baixas taxas de CS (18.4 e 16.9%, respectivamente).

**Conclusão** O RTGC é uma ferramenta útil para avaliar as taxas de CS em diferentes unidades de saúde. Os grupos 5, 1 e 3 foram os principais contribuintes para a taxa de CS. Estes achados podem apoiar intervenções futuras para reduzir as CS desnecessárias, especialmente entre primíparas e em mulheres com uma CS anterior.

## Introduction

In the last decades, a progressive increase in the global rates of cesarean section (CS) has been reported, which does not correlate with a decrease in maternal and neonatal adverse outcomes. Therefore, the World Health Organization (WHO) updated its recommendations in 2014, confirming previous reports that CS rates > 10 to 15% are not associated with a reduction in maternal morbidity and mortality. 1 These rates, however, might be currently not achievable in many settings and in different countries, and recent studies have attempted to consider underlying differences. 2

The CS rates in the United States of America reached 32%. 3 In Latin America and in the Caribbean, the reported CS rate was 40.5% in 2014, with higher rates in Brazil (55.6%) and in the Dominican Republic (56.4%). South America reported an overall 42.9% of CSs, which is among the highest rates worldwide. 4 To understand CS rates in each setting, the WHO adopted an easy and reliable classification system called the Robson Ten Group Classification (RTGC), based on obstetric characteristics at admission for childbirth (parity, gestational age, onset of labor, previous CS, fetal presentation, and the number of fetuses). 5

The RTGC has been implemented in several settings; however, few reports from Central America are available. Honduras is a low-income country with almost nine million inhabitants and a human development index (HDI) of 0.623. 6 7 According to official records from the Ministry of Health, CS rates increased in Honduras from 25.8 to 35.8% in the last decade. Considering the lack of reliable information about the causes of this increase, our aim is to use the RTGC in all women admitted for childbirth during a 15-month period in a referral center in Honduras, to evaluate the overall distribution among groups and the contribution of each group to the CS rate, with special focus in groups that consider induction of labor.

## Methods

This was a cross-sectional study approved by the local Ethics committee (#20112019). All women admitted for childbirth (≥ 22 weeks) at the Hospital Roberto Suazo Cordova, La Paz, Honduras, between August 1, 2017, and October 31, 2018, were considered. The Hospital Roberto Suazo Cordova is a public hospital that provides antenatal and childbirth care for low- and high-risk women, with ∼ 3,600 deliveries per year, 20 hospital beds for obstetric care, no intensive care beds, and a low maternal mortality rate, with no maternal deaths in the considered period.

Initially, the overall CS rate was calculated. Then, all women were stratified according to the RTGC, which consists of data on obstetric history (nulliparity, multiparity), previous CS, onset of labor (spontaneous, induced, or CS without labor), number of fetuses (single or multiple), and situation and presentation (cephalic, breech, and transversal). Three of the 10 groups (Group 2, 4, and 5) were further subdivided and analyzed based on the criteria proposed by Robson: onset of labor for Groups 2 and 4; and the number of previous CSs the patient had for Group 5. Therefore, Group 2 was divided in subgroup 2a (all nulliparous with single cephalic pregnancy, ≥ 37 weeks with induction of labor) and Sub-group 2b (all nulliparous women with single cephalic pregnancy; ≥ 37 weeks with CS before labor). Group 4 was also divided in subgroup 4a (all multiparous women without previous uterine scar, with a single cephalic pregnancy ≥ 37 weeks, who underwent induction of labor), and subgroup 4b (all multiparous women without previous uterine scar, with a single cephalic pregnancy ≥ 37 weeks, who underwent CS before labor); and Group 5 was divided in subgroup 5.1 (all multiparous women with 1 previous uterine scar with a pregnancy ≥ 37 weeks, in cephalic presentation) and subgroup 5.2 (all multiparous women with a history of ≥ 2 previous uterine scars with pregnancy ≥ 37 weeks, in cephalic presentation). This subdivision allows for the assessment of the impact of induction of labor and of elective CS on the CS rates and the contribution of vaginal birth after cesarean section (VBAC).

To retrieve these data, we used the standard data collection system implemented in our hospital and used throughout many Latin American countries, called Perinatal Information System (SIP, in the Spanish acronym) version 4.16, from the Pan-American Health Organization/World Health Organization/Centro Latino-Americano de Perinatologia (PAHO/WHO/CLAP). The SIP is used for standard healthcare in obstetrics and gynecology. The SIP database includes the obstetric and clinical history, the antenatal follow-up chart, and the partograph, as well as maternal and perinatal outcomes. All the data are entered in the medical chart by health professionals, and it is subsequently included in the SIP by trained personnel. 8

The classification of the women in the different groups and the CS rates were obtained using an option already available in the SIP program called “INFORM – ROBSON INDICATORS” that allows the program, using previously selected variables, to classify the women in the 10 groups presenting their relative size, the CS rate by group, and the contribution of each group to the global CS rate. To confirm the consistency of this output, 10% of the sample (436 cases) were also classified manually (by review of medical charts) considering all the needed variables for the RTGC. Since the difference in results between information obtained by the SIP system and those obtained manually corresponded to only 1 case (0.02%), the results of the SIP databased were considered consistent and further used.

To determine the relative size of each group of the Ten Group Classification System (TGCS), the frequency of the groups was determined, calculated by dividing the total number of women in each group by the total of women included in the study. The contribution of each group to the overall CS rate was also analyzed using the frequency of CS per group, calculated dividing the total number of CSs in each group by the total of CSs during the study period. The main clinical indications for CS were considered for all women that had a CS as route of delivery.

Sociodemographic characteristics (age, educational level, and marital status) and maternal outcomes (hemorrhage, hypertension, and infection) were compared among women with vaginal birth and CS as a supplementary file. Infection included chorioamnionitis, endometritis, surgical wound infection, and sepsis; hypertensive disorders included gestational hypertension, pre-eclampsia (PE), chronic hypertension, and superimposed PE; and hemorrhage comprised bleeding complications in the 2 ^nd^ and 3 ^rd^ trimesters and in the postpartum).

A descriptive analysis was performed using Epiinfo version 7.2 (Centers for Disease Control and Prevention), and for the comparison among groups, the chi squared uncorrected test and the t-Student test were used, considering a p-value < 0.05 statistically significant.

The clinical protocols of the institution were considered to interpret the results. The considered maternity hospital does not perform induction of labor in women with a history of one previous CS.

## Results

**Fig. 1:**
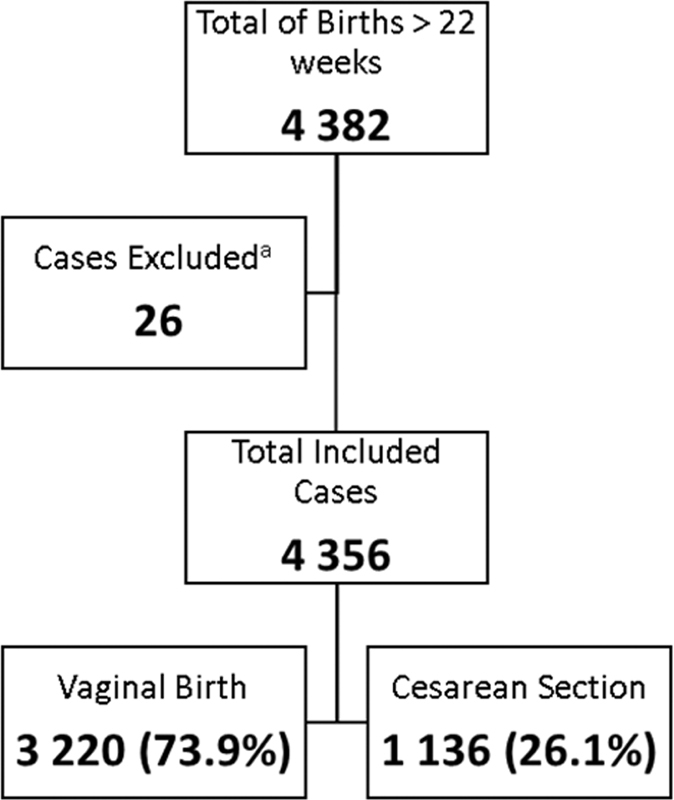
Flowchart of Included cases. (a) Women were excluded for missing data on both medical chart and electronic medical chart.

The total number of deliveries in the period was 4,382; of these, 26 women were excluded due to missing information, with a total of 4,356 included cases. The overall CS rate was 26.1% ( Fig. 1 ). The distribution of women based on the RTGC, the specific rate of CS per group, and the contribution to the overall rate of CS is presented in Table 1 with a description of each group. Most women admitted for childbirth during the study period were from Group 3 (1,682/4,356 (38.6%); followed by Group 1 with 1,342/4,356 (30,8%); and by Group 5 with 450/4,356 (10.3%). Groups 6 to 10 presented the lowest number of women per group and the lowest contribution to the overall CS rates, as presented in Table 1 .

**Table 1:** Rate of cesarean delivery by the Robson Ten Group Classification System among women admitted for childbirth, cesarean section rate, relative size of groups, cesarean section rates by group, and contribution of each group to the overall cesarean section rate ( *n* = 4,356)

GROUP	GROUP DESCRIPTION	RELATIVE SIZE OF THE GROUP % ( *n* )	CS RATE WITHIN GROUP % ( *n* )	ABSOLUTE GROUP CONTRIBUTION TO OVERALL CS RATE % ( *n* )	RELATIVE GROUP CONTRIBUTION TO OVERALL CS RATE % ( *n* )
1	Nulliparous, single, cephalic, ≥ 37 weeks, spontaneous onset	30.8% (1,342/4,356)	21.7% (291/1,342)	6.7% (291/4,356)	26.62% (291/1,136)
2	Nulliparous, single, cephalic, ≥ 37 weeks, induced onset or elective CS	6.2% (268/4,356)	38.8% (104/268)	2.4% (104/4,356)	9.15% (104/1,136)
3	Multiparous, without cesarean delivery scar, single, cephalic, ≥ 37 weeks, spontaneous onset.	38.6% (1,682/4,356)	9.1% (153/1,682)	3.5% (153/4,356)	13.47% (153/1,136)
4	Multiparous, without a cesarean delivery scar, single, cephalic, ≥ 37 weeks, induced onset, or elective CS	5.0% (216/4,356)	25.0% (54/216)	1.2% (54/4,356)	4.75% (54/1,136)
5	All multiparous women with at least 1 previous CS, single, cephalic, ≥ 37 weeks.	10.3% (450/4,356)	76.7% (345/450)	7.9% (345/4,356)	30.37% (345/1,136)
6	All nulliparous, single, breech	0.9% (40/4,356)	87.5% (35/40)	0.8% (35/4,356)	3.08% (35/1,136)
7	All multiparous, single, breech, includes women with a previous CS	1.4% (61/4,356)	78.7% (48/61)	1.1% (48/4,356)	4.23% (48/1,136)
8	All women with multiple pregnancy, includes women with a previous CS	1.1% (48/4,356)	64.6% (31/48)	0.7% (31/4,356)	2.73% (31/1,136)
9	All women, single, in transverse or oblique, includes women with a previous CS	0.7% (31/4,356)	100% (31/31)	0.7% (31/4,356)	2.73% (31/1,136)
10	All women, single, cephalic, ≤ 36 weeks, includes women with a previous CS	5.0% (218/4,356)	20.2% (44/218)	1.1% (44/4,356)	3.87% (44/1,136)
TOTAL			26.1% (1,136/4,356)		100% (1,136)

Abbreviation: CS, cesarean section.

Considering the contribution of each group to the CS rate, Group 5 (multiparous women with previous CS and pregnancy ≥ 37 weeks) represented 30.4%; Group 1 (nulliparous women with single pregnancy ≥ 37 weeks and spontaneous onset of labor) 26.6%; and Group 3 (multiparous women with single pregnancy ≥ 37 weeks and with spontaneous onset of labor) 13.5% ( Table 1 ).

Groups 2, 4, and 5 of the RTGC were divided into 2 subgroups each – Groups 2 and 4 considering the onset of labor (induction defines Groups 2a and 4a); and Group 5 considering the number of previous CSs (1 previous scar defines 5.1). In Group 2, three-quarters of the women corresponded to subgroup 2a, 201/268 (75%); when analyzing the CS rate of subgroup 2a, we found a low rate (18.40%; 37/201), indicating a high success rate of labor induction among nulliparous women. Group 4 had ∼ 90% (195/216) of the cases within subgroup 4a, and a greater success of induction of labor with a CS rate of 16.92% (33/195) among induction cases ( Table 2 ). A total of 450 women had a history of at least 1 previous CS (Group 5), most of them in subgroup 5.1 (86.9%; 391/450), with a CS rate of 73.15% (286/391), and subgroup 5.2 represented 13.1% (59/450) of the women within Group 5, with a CS rate of 100% ( Table 2 ), since it is the protocol of the institution to perform scheduled CS in cases with ≥ 2 previous CS.

**Table 2:** Distribution of cases among subgroups of the Ten Group Classification System and cesarean section rate within groups

GROUP	DESCRIPTION	TOTAL NUMBER OF CASES PER GROUP	SUBGROUP *n* (%)	SUBGROUP DEFINITION	CS RATE WITHIN THE SUBGROUP % ( *n* )
2	Nulliparous women with single cephalic pregnancy ≥ 37 weeks, who underwent induction of labor or CS before the start of labor	268	2a 201 (75%)	Induced labor	18.40% (37/201)
			2b 67 (25%)	Scheduled CS (prior to labor)	100.00% (67/67)
4	Multiparous women without previous uterine scar, with a simple cephalic pregnancy ≥ 37 weeks, who underwent induction of labor or CS before the start of labor	216	4a 195 (90.3%)	Induced labor	16.92% (33/195)
			4b 21 (9.7%)	Scheduled CS (prior to labor)	100.00% (21/21)
5	All multiparous women with a history of at least 1 previous uterine scar with pregnancy ≥ 37 weeks, cephalic	450	5.1 391 (86.9%)	One previous CS	73.15% (286/391)
			5.2 59 (13.1%)	≥ 2 previous CSs	100.00% (59/59)

Abbreviation: CS, cesarean section.

The main reported indications for CS were a previous CS (30.8%; 350/1136), acute fetal distress (24.5%; 278/1136), and labor arrest (17.8%; 202/1,136). The RTGC does not consider clinical background or maternal complications; nevertheless, outcomes are always an important finding in comparing CS and vaginal deliveries, and we performed this analysis (supplementary file). Regarding obstetric characteristics, the findings were similar in both groups. When comparing sociodemographic characteristics, there was a statistically significant difference in maternal age and educational level, with more CS among older and more educated women. Comparing the main maternal complications, hypertensive disorders were the only significantly associated complication with an increased frequency among the CS group ( *p <* 0.0001).

## Discussion

The present study evaluated a 15-monthperiod at a maternity hospital in Honduras using the available database implemented for healthcare (SIP) to retrieve information on the RTGC and analyze CS rates. The overall CS rate was 26.1%, with Groups 5, 1 and 3 the ones that presented the highest contribution to the overall CS rate. The subgroup analysis presented a high successful rate of induction of labor for Groups 2a and 4a.

The CS rate presented does show an increase from official data on Honduras, and also within the considered institution, who reported 15.9% a decade ago (unpublished data retrieved from the SIP database). However, when comparing with numbers from Latin America, it is still one of the lower CS rates in the region. 2 3 4

When analyzing the groups with the greatest contribution to the overall CS rate, our results are in agreement with other studies worldwide, in which multiparous women with a history of at least 1 previous CS, gestational age ≥ 37 weeks, and in cephalic presentation (Group 5) are among the main contributors, 9 ranging from 17.2% in South Africa 10 11 12 13 to 35.6% of all CSs in Colombia. 10 These findings suggest that interventions to reduce the CS rate need to be implemented in Group 5, aiming to ascertain a VBAC; nevertheless, at the same time, the most important action should be preventing the first CS in women of Groups 1 to 4. 10 11 12 13 14

The decision to perform a CS is influenced by many factors within each setting, including social, economic, and cultural background, private versus public institutions, risk, or awareness of litigation, for example. All of these must be considered. 15

In 2013, Robson et al. published a study with the suggested ideal CS rate for each group of the RTGC. 16 When comparing these rates with our study, the findings from Groups 1, 3, and 5 were above the suggested CS rate. The suggested rates for each group were defined as: < 10%, < 3%, and 50 to 60%, respectively. Analyzing the CS rate within Group 5, we found in our study that the rates were > 70%, significantly above the suggested by Robson et al. The total number of cases in Group 5 represented ∼ 10% of the overall women considered. According to Robson et al., when the size of this group is < 10%, it “reflects a previous low CS rate. If higher, there has been a high caesarean section rate in the past years, mainly from groups 1 and 2.” 16 Our findings among Group 5 are also a consequence of the protocol of the institution, with no induction of labor in cases of one previous CS. Among these cases (subgroup 5.1), either women are followed until spontaneous onset of labor or scheduled for a CS. It is important to consider that complications associated with VBAC are rare when adequate intrapartum care is provided. 17 18 19 Based on the interpretation of the TGCS suggested by Robson, 16 the presence of a CS rate for Group 2 > 35% suggests a high rate of prelabor CS. Our results did not present this finding, with low CS rates among groups 2 and 4. These findings support those interventions must be implemented in Groups 1 to 4 to prevent the first CS, avoiding further impact on an increase of the overall CS rate. 13 20 21 22

The strategies for the prevention of CS in nulliparous women can be considered based on for whom they are aimed: women or health care professionals. Possible intervention toward women could be the implementation of Childbirth Training Workshops or the improvement of healthcare and information during medical visits. 23 24 Increasing women's knowledge of what to expect during childbirth and improving both the labor experience and the outcomes could help decrease CS and increase spontaneous vaginal deliveries. Therefore, providing analgesia for vaginal delivery, avoiding early admission, providing respectful care, and allowing companionship are also important interventions. Conducting audit and feedback in combination with the implementation of medical practice guidelines or requesting a second opinion to confirm the need for a CS could be some of the possible interventions for healthcare providers. 23 24 25

Through the subdivision of Groups 2 and 4 according to the parameters proposed by the authors of the RTGC (induction of labor or elective cesarean delivery), we can perform an analysis of the success rate of the induction of labor with oxytocin and misoprostol induction protocols used in the Hospital Dr. Roberto Suazo Cordova. We found a high success of induction of labor for both subgroups 2a and 4a, with a success of induction of labor in 81.6% of the women in subgroup 2a and a higher success of induction of labor (83.08%) for subgroup 4a (multiparous women). This success rate was similar to the rates reported by Deshmukh et al., who reported a success rate of 80.5% for vaginal delivery within 24 hours of induction with oral misoprostol. 26

In Honduras, the medical guideline “Normas Nacionales para la Atención Materna-Neonatal” (National Guideline for Maternal-Neonatal care) has been implemented as a standard protocol for maternal and neonatal care. These guidelines include recommendations for labor induction; however, for women with previous CS in Honduras, expectant management is suggested, without any type of induction of labor. 27

Considering that Group 5 is the largest contributor to the CS rate and that we reported very good results among induction of labor in subgroups 2a and 4a, a future intervention should be implemented for the introduction of protocols for cervical preparation and induction of labor in cases of 1 previous CS. In a previous study by Gobillot et al., 28 a success rate for vaginal birth > 80% in VBAC was reported with induction of labor using oxytocin, and a rate of uterine rupture of 3% in the induction group. An important aspect of induction of labor in women with a previous CS is that this decision must be performed jointly between the treating obstetrician and the woman after she is properly informed of the advantages of vaginal deliveries and of the possible complications or risks. It has been reported in different studies that the mechanical induction of labor with a balloon catheter for cervical ripening is an induction mechanism that can be used safely in women with a history of uterine scar and with an acceptable success rate. Sarreau et al. 29 reported that 38.4% (58/151) of the women initiated labor before the removal of the balloon catheter, with 75% (42/58) of vaginal births. Rossard et al. 30 reported that using a balloon catheter for cervical ripening improved Bishop scores before induction of labor in women with a previous CS, with vaginal delivery in 64.1% of the women with a previous CS. Results comparable to those of the aforementioned studies are found in the literature considering induction of labor among women with one previous CS, achieving vaginal delivery in between 71 and 80% of the cases. 31 32 Keeping in mind that Honduras, at the moment, does not have a protocol for induction of labor in women with a previous CS, the future implementation of such a protocol should be considered one of the key interventions to help decrease the CS rates in Group 5.

To plan specific interventions, it is key to further detail findings in Groups 2b, 4b, and 5.1. Understanding the underlying reasons for a scheduled CS and looking into cases of one previous CS to see if trail of labor after CS or VBAC were attempted; unfortunately, at this moment, our study was limited by the information provided by the SIP database and these data were not available.

The SIP has proven to be a useful tool for data collection by providing a complete database that is easy to access and has a friendly interface. The most important limitation observed in the SIP was the inadequate filling by the healthcare professional, which leads to incomplete information in the database, limiting the depth of the analysis when considering maternal and perinatal outcomes by group and even some sociodemographic characteristics that might be interesting for comparison and to complement findings on the RTGCS. The main criticism of this classification is that it does not consider underlying comorbidities or maternal complications. That was the rationale for the presented comparison in our supplementary file; however, we must acknowledge that it was difficult to retrieve detailed data on selected outcomes and we were able to compare CS and vaginal deliveries with no comparisons among Robson groups. Another limitation during the study was not being able to properly analyze the main indication of CS for Groups 2 and 4.

To plan adequate interventions and discuss CS rates in different settings, we must consider overall outcomes on maternal mortality and morbidity, which are not included in the RTGC. The studied institution in Honduras had no maternal deaths in the study period and when comparing the main causes of maternal morbidity 33 34 : hypertension, hemorrhage, and infection, among cases of vaginal birth and CS; the only significant difference was on the frequency of hypertensive disorders, with more cases among the CS group. Other studies report an increased CS rate among cases complicated by hypertension/(PE, especially among cases of severe disease and early-onset PE. 35 The relevance os PE is also considered in the C-model, a calculator developed to provide information on C-section probability. The C-model considers not only the information from RTGC, but also data on demographics (maternal age) and severity (organ dysfunction or Intensive Care Unit admission) and complications (including PE). 2

The use of the RTGC among women admitted for childbirth can allow the identification of the main obstetrics characteristics of the population served by health facilities. It also allows the understanding of the characteristics associated with an increase of CS rate in different health facilities, and the correct interpretation of the information provided by the RTGC can enable specific interventions to reduce the CS rate. This information can be subsequently shared and/or compared with other health facilities or settings worldwide. The strength of the present study is showing how an implemented database could be used, even prospectively, to report not only on overall CS rates, but already considering the RTGC. The SIP database is available in ∼ 34 countries in Latin America and the Caribbean 36 and the same analysis reported here can be promptly performed in all these settings.

## Conclusion

The RTGC is a useful tool to assess CS rates in different healthcare facilities and the SIP database can retrieve this information. Groups 5, 1, and 3 were the main contributors to the CS rate and Groups 2 and 4 demonstrated the impact and importance of induction of labor. These findings may support future interventions to reduce unnecessary CSs, especially among primiparous women and in women with a previous CS.
